# Understanding Workplace Violence in ED: The Maternity Nurses’ Experience Using Descriptive Phenomenology

**DOI:** 10.1155/jonm/6649762

**Published:** 2026-06-23

**Authors:** Alia Serour Ahmed, Glenita Castelino, Ruth De Leon, Lali Paulose, Sindhu Mathew, Sigi Ann Varghese, David Hali De Jesus, Ruby Raj Mariyamma, Albara Mohammad Ali Alomari, Rana Aatif Salim Ibrahem, John Paul Ben Silang

**Affiliations:** ^1^ Nursing and Midwifery Department, Women’s Wellness and Research Center, Hamad Medical Corporation, Doha, Qatar, hamad.qa; ^2^ College of Health Sciences, University of Doha for Science and Technology, Doha, Qatar; ^3^ Clinical Research Unit in Nursing and Midwifery, Department of Research, Women’s Wellness and Research Center, Hamad Medical Corporation, Doha, Qatar, hamad.qa

**Keywords:** emergency, maternity, nurses, tertiary care, workplace violence

## Abstract

**Introduction:**

Workplace violence (WPV) in clinical settings is a global issue in healthcare. Recent studies have shown that attitudes toward WPV are evolving. According to the World Health Organization, WPV affects between 8% and 38% of healthcare workers, especially in culturally diverse nations like the Middle East. The emergency department (ED), as one of the busiest and most critical hospital units, has been reported as the most common focal point for WPV. Thus, this study describes the WPV experiences of maternity nurses and emphasizes the importance of understanding this phenomenon to inform effective interventions.

**Methods:**

The study followed a descriptive phenomenology approach. Fifteen ED nurses were selected and interviewed with a semistructured interview guide and an audio recorder. Participants were recruited via telephone invitations based on confirmed WPV reports. One‐on‐one interviews were conducted until data saturation was reached. Audio recordings were transcribed, and data were analyzed using Colaizzi’s thematic analysis method, ensuring trustworthiness and rigor in accordance with Lincoln and Guba’s criteria.

**Results:**

Data analysis identified 10 subthemes and 5 emerging themes describing maternity nurses’ WPV experience in a tertiary care setting. Themes include demanding emergency care through verbal aggressiveness; sociocultural differences influencing nurse‐patient interaction and emergency nursing care; misaligned expectations and policy awareness; nurses’ adaptation and competing care demands; and managing WPV with manpower resources and collaboration.

**Conclusion/Recommendations:**

The study reveals that WPV has multiple drivers: system‐based, situation‐grounded, and contextual. This evidence, along with the body of current literature, supports the plea to have a zero‐tolerance policy for WPV, especially in maternity settings where the safety of the woman and her child is the utmost priority.

## 1. Introduction

Workplace violence (WPV) in healthcare refers to any act or threat of physical harm, harassment, or intimidation occurring in the workplace against healthcare professionals, including nurses [1]. It is globally recognized as a serious occupational and public health concern. Both verbal and physical abuse are acknowledged as occupational hazards in hospital settings [2]. The emergency department (ED) is particularly vulnerable, reporting some of the highest rates of WPV across healthcare systems [3]. The most frequently reported sources of violence are patients and their family members [4].

Research consistently shows that WPV in EDs is linked to factors such as inadequate staffing, lack of privacy, overcrowding, and unsecured medical equipment that may be used as weapons [5]. Nurses, as frontline caregivers, experience high levels of verbal and physical aggression [6], and even student nurses report exposure to various forms of abuse [4]. This persistent exposure has been associated with increased psychological distress, decreased job satisfaction, and reduced quality of care [7, 8]. Despite extensive research, there remains no clear consensus on the most effective strategies for preventing and mitigating WPV [9].

In maternity and emergency settings, particularly within multicultural healthcare systems, WPV may be influenced by complex emotional, cultural, linguistic, and legal factors [10]. Multiple studies from Arab countries, including Sudan [11], Egypt [12], Saudi Arabia [13], and Jordan [14], consistently reported a high prevalence of WPV against nurses, with verbal and psychological abuse emerging as the most common forms. These regional findings highlight shared challenges across Arab healthcare systems, where patient relatives often act as perpetrators and under‐reporting remains a persistent issue. Given these common patterns, there is a growing need to explore how emotional, cultural, and linguistic factors shape the occurrence and management of WPV.

Support for healthcare workers in managing WPV must be multifaceted. A holistic approach that integrates peer‐support programs and encourages sharing of real‐life experiences is essential [15]. Such insights can guide institutions toward timely and practical solutions. A participatory, interprofessional approach engaging nurses, physicians, security personnel, and administrators is recommended for developing and implementing anti‐violence strategies. According to the WHO, effective collaboration improves communication, facilitates the early identification of risk factors, and strengthens workplace safety protocols [10]. This approach is particularly valuable in multicultural environments such as Qatar, where diverse languages and cultural norms influence staff–patient interactions. Qatar is home to a highly diverse population, and its first tertiary obstetrics and gynecology hospital plays a critical role in promoting women’s health and well‐being. The ED of the selected maternity facility is a Level II service that handles approximately 7000 patients each month, making it one of the busiest units. As the primary point of patient admission, particularly during bed shortages, the study site is among the departments most at risk for WPV. Within this 29‐bed unit, staffed by around 100 ED nurses, the staff provide initial treatment for patients with obstetric, gynecologic, and newborn health conditions. There are relevant cases, maybe life‐threatening, that require immediate action, in which nurses work collaboratively with other healthcare specialists. Depending on clinical findings, patients are either treated and discharged directly from the ED or admitted to the inpatient obstetric units for further management. Several training sessions and educational programs on WPV have been conducted, including conflict resolution and the prevention and management of violence and aggression, to which frontline nurses are invited to participate.

At the study site, the incidents of verbal and physical abuse from patients and their relatives have been recorded through the occurrence, variance, accident (OVA) reporting system. After OVA reporting, the affected staff receives feedback about the incident. Data showed that 46% of reported WPV incidents came from the ED, but this figure accounted for only 0.4% of the total patient encounters. Under‐reporting of WPV often occurs when such incidents are perceived as normal or a common experience when working in the ED. Other factors contributing to under‐reporting include fear of retaliation, a lack of action plans within the support system, and undefined criteria for what constitutes a reportable WPV‐related incident. The incidents reported in this study fall under Type II violence, as classified by the National Institute for Occupational Safety and Health (NIOSH), where patients or visitors become violent during the care process. The hospital maintains a zero‐tolerance policy for any form of violence, aggression, or abuse directed at staff members. All patients and visitors are expected to behave respectfully toward healthcare professionals and others within the facility. When a patient or visitor displays verbally abusive, physically aggressive, or threatening behavior, two primary response protocols are implemented according to the severity of the incident. The rapid response team, accessible via a dedicated hotline and trained in conflict management, addresses violent incidents and completes required reporting. If the behavior continues or presents an ongoing threat, security personnel intervene to manage or remove the individual from hospital premises. In cases of severe incidents, such as assaults, threats, or property damage, security personnel notify the police, which may result in legal action [16].

In Qatar, all maternity ED nurses are female, reflecting the country’s Islamic sociocultural context, which emphasizes women’s safety and explicitly prohibits physical abuse [17, 18]. However, verbal abuse remains the most common form of WPV, followed by psychological and physical assault [19]. Common antecedents to WPV include high emotional distress among patients and families experiencing fear, anxiety, and frustration. Public expectations, including those of many patients and attendants, often reflect a limited understanding of triage systems and may lead to delays being perceived as neglect. The maternity ED caters to both local and expatriate populations, with a continuous influx of obstetric emergencies and early pregnancy complications, which leads to long waiting times and high patient volume. Previous studies have identified long waiting times, understaffing, and miscommunication as key triggers for verbal aggression [20]. Threats, intimidation, yelling, and insults are typical forms of verbal aggression experienced regularly, ranging from once every few days to once a week [21]. Another study highlighted the widespread nature of WPV among nurses in Qatar [22]. The maternity nurses in ED represent a particularly critical group to examine, as they work in demanding, high‐pressure environments where exposure to verbal and physical abuse is frequent, yet their experiences remain underexplored. This study addresses that gap by adopting a descriptive, qualitative design to explore the lived experiences of maternity ED nurses through in‐depth interviews. By exploring the lived experiences of maternity ED nurses, this study moves beyond numerical data to reveal the meaning and its essence WPV within the clinical context. Ultimately, the findings aim to enhance understanding of maternity ED nurses’ experiences and provide evidence‐based recommendations to prevent and respond to WPV.

## 2. Aim

To describe the lived experience of maternity ED nurses exposed to WPV and unravel the essence of the phenomenon. Research question: “*What is the essence of WPV as experienced by maternity ED nurses?*?”

## 3. Methodology

### 3.1. Study Design

The study followed the qualitative research design grounded in Husserl’s descriptive phenomenology, aiming to describe the lived experiences of maternity ED nurses and unravel the essential structure of WPV. Husserlian phenomenology guided data collection and analysis through epoché (bracketing), descriptive reduction, and focus on the essential structures of participants’ experiences. This approach allowed investigators to capture nurses’ awareness of their environment, sense of self, and interactions within the workplace. The primary goal was to gain a deeper understanding of WPV in a selected tertiary maternity facility in Qatar. The study adheres to the Standards for Reporting Qualitative Research (SRQR) to ensure transparent and rigorous reporting of design, sampling, data collection, analysis, and trustworthiness.

### 3.2. Recruitment of Participants

To ensure confidentiality, the Nurse Midwife Research Scientist (NMRS) was responsible for selecting, recruiting, interviewing, and transcribing the data. The NMRS requested the OVA reports relevant to WPV in ED for the period of 2019 to 2020. Thus, the WPV incident occurred within the past 12 months. The request was addressed to the Quality and Patient Safety (QPS) Department, which is responsible for documenting these reports at the study site.

Nurses were selected based on the following specific eligibility criteria: (1) nurses who had experienced WPV based on the QPS reports from 2019 to 2020, (2) licensed nurses by DHP, (3) those who had completed the six‐month probation period, (4) currently working in the ED of the selected maternity facility, and (5) English‐speaking. Purposive sampling was used for selection. Nurses who did not work in the ED or had no WPV experience were excluded.

Fifteen nurses were selected from the list based on eligibility criteria. The selected participants were invited to take part in one‐on‐one interviews with the NMRS by phone. Those who confirmed participation were assigned participant codes (e.g., P1, P2, and P3) to maintain anonymity. The codes linking to the participants were recorded on the subject code log sheet and stored on the NMRS desktop, protected by username and password.

### 3.3. Data Collection

The interviews were conducted by the NMRS, which has no direct managerial authority over the respondents. To ensure privacy, the interviews took place in an interview room at the facility’s Research and Education Center. Each interview session lasted 20 to 60 min. An audio recorder was used, and the data were transcribed by the NMRS using Microsoft 365. The interviews were conducted individually and face‐to‐face. Key points and valuable responses from participants during the interview were also documented. The interviews with the 15 eligible participants were conducted in series, and saturation was reached in the fifth session.

### 3.4. Data Collection Tool

A semistructured interview guide was developed to explore maternity ED nurses’ perceptions and lived experiences of WPV. The guide was designed in accordance with Husserl’s descriptive phenomenology, emphasizing participants’ subjective meanings and reflections on their experiences. It was content validated by qualitative research experts to ensure clarity and relevance. A pilot interview was conducted to assess comprehensibility and sequencing, and minor refinements were made accordingly. Questions were open‐ended and flexible, allowing participants to elaborate freely and ensuring depth of insight (see Appendix A).

### 3.5. Data Analysis

Two doctorate‐level investigators with expertise in thematic analysis reviewed the data and derived themes using Colaizzi’s method, a rigorous approach that ensures credibility and reliability in qualitative research [23]. The process involved: (1) repeatedly listening to audiotapes for familiarity, (2) examining transcripts line‐by‐line for accuracy while highlighting and coding key statements, (3) categorizing and clustering codes into themes, and (4) using these themes to capture the essence of experiencing WPV. This method effectively revealed emergent themes and their interwoven relationships, ensuring a robust qualitative analysis. Data saturation was achieved on the fifth session from the selected 15 voluntary participants, as codes and categories became repetitive and no new themes emerged.

### 3.6. Ethical Considerations

The project was reviewed and approved by the Institutional Review Board of the Medical Research Center of Hamad Medical Corporation in Doha, Qatar (MRC No. 01‐21‐817). Participation in the study was entirely voluntary. Thus, they were verbally informed and, in the informed consent, stated that they could decline to participate without consequences. Moreover, they were also informed about the purpose of the research, the significance of the findings, and their rights as participants. Informed consent was obtained on the day of the interview, and then, the interview questions were asked. After the first session, follow‐up questions were used in subsequent interview sessions until data saturation was reached.

### 3.7. Trustworthiness and Rigor

The process for evaluating the reliability and validity of the research findings was adapted from Lincoln and Guba’s four‐dimension criteria (FDC) for trustworthiness in qualitative research [24]. In this study, two subject‐matter or content experts were invited to work with the investigators to review relevant research files, including both methodology and findings. The two experts, who hold doctoral degrees and have experience in qualitative research, were asked to evaluate the study’s trustworthiness and rigor. Two criteria were reviewed for the validity of the research findings. The first criterion is credibility, which was assessed through member checking, with results shared to obtain feedback on whether the analysts’ findings accurately represented and resonated with the participants’ lived experiences. Second is transferability, whereby the themes and details of the findings were presented with rich, thick descriptions to ensure the applicability of the study results to similar settings. For reliability, dependability was used as a criterion to evaluate whether the processes and procedures are auditable. Hence, an audit trail was used to conserve the methods and findings and ensure their transparency and traceability. For objectivity or confirmability, the aim is to strengthen confidence that the findings remain consistent between data analysts. Thus, two research experts examined the theme derivations until both confirmed their judgments about findings. Any ambiguities were clarified, thereby enhancing confirmability and ensuring the study’s rigor.

## 4. Results

### 4.1. Profile

As shown in Table 1, the majority of the 15 participants were aged 31–40 years (67%) and held a bachelor’s degree (87%). Around 73% were employed as Graduate Registered Nurses, while 20% were staff nurses. Additionally, the findings also showed that approximately 73% have been working as nurses for more than 10 years, and about 60% have 6–10 years of working experience in the ED of the selected facility.

**TABLE 1:** Profile characteristics of the participants.

Variables	Frequency	Percentage
Age		
20–30 years	0	0%
31–40 years	10	67%
41–50 years	4	27%
> 50 years	1	6%
Education level		
Diploma	2	13%
Bachelor’s degree	13	87%
Postgraduate degree	0	0%
Job position		
Staff nurse (diploma nurses)	3	20%
Graduate registered nurse	11	73%
Charge nurse	1	7%
Duration of clinical experience as a nurse		
1–5 years	0	0
6–10 years	4	27%
> 10 years	11	73%
Duration of work experience in the maternity ED in the selected facility		
1–5 years	2	13%
6–10 years	9	60%
> 10 years	4	27%

### 4.2. Themes

To understand WPV in the maternity ED, narratives from 15 nurses were analyzed, yielding five themes and 10 subthemes clustered from over 67 initial and final codes (see Table 2).

**TABLE 2:** Themes, subthemes, and meaning of the WPV experience as described by staff nurses in the maternity ED.

Themes	Subthemes	Meaning
1. Demanding Emergency Nursing Care Through Verbal Aggression	*Demand-based interaction*	The patients and their relatives express aggression verbally by raising their voice and making offensive statements in response to unmet expectations and perceived urgency for urgent nursing care. It is the means by which they influence nurses to deliver emergency nursing care promptly. It also indicates a mismatch between nurses’ perceived needs and those of patients and their relatives, leading to dissatisfaction with nursing care outcomes.
	*Dissatisfaction with the outcomes of care*	

2. Sociocultural Differences Influence Nurse–Patient Interaction and Emergency Nursing Care	*Language as a barrier to mutual understanding*	The theme highlights gaps in communication arising from language and cultural differences. Communication influences nurse–patient interactions, and when it fails, it often leads to conflict and WPV. However, themes also indicate that a diverse workforce is an essential manpower resource as it provides culturally and linguistically aligned nurses. Staff diversity addresses the consequences of sociocultural differences in emergency nursing care. Having representation can shape better nurse–patient interactions, resolving conflicts, and mitigating WPV.
	*Diverse representation resolves conflict*	

3. Misaligned Expectation and Policy Awareness	*Nonadherence due to limited awareness of the hospital guidelines*	A lack of awareness of policies and procedures affects how patients and their relatives perceive, behave, and respond to nurses in the ED. Thus, prioritization, delays, restrictions, and privileges in the ED are interpreted based on their understanding and experience. The misalignment between nurses’ perceptions of emergency nursing care and those of patients and their families, shaped by limited awareness, can lead to potential conflict and WPV.
	*Patients’ and families’ expectations override hospital policy*	

4. Nurses’ Adaptation and the Competing Care Demands	*System pressure from competing demands*	Maternity emergency care functions within a high‐pressure environment. It is characterized by competing care demands and a mismatch between nurses’ and patients’ perceptions of emergency nursing care needs. This pressure creates a challenging work environment that is unfavorable to both nurses and patients, leading to conflict and WPV. However, nurses adapt and employ coping strategies that sustain emergency nursing care and manage workflow interference and urgency.
	*Resilience of the emergency nurses*	

5. Managing WPV with Manpower Resources and Collaboration	*Workforce crisis increases risk to WPV*	Staff shortages lead to high workloads and delays in care delivery, which can trigger frustration among nurses, patients, and their families, thereby increasing the likelihood of WPV. Collaboration with nurses and other ED staff, de‐escalation of aggression, and promotion of safety. With a team, nurses feel supported, which can help them regulate emotions and sustain work.
	*Team-based response for safety and managing aggression*	

#### 4.2.1. Theme 1: Demanding Emergency Nursing Care Through Verbal Aggression

Verbal aggression emerged as the predominant form of WPV experienced by maternity nurses in the ED, often arising from patients’ and relatives’ unmet expectations and demands.

##### 4.2.1.1. Subtheme 1.1. Demand‐Based Interaction

The demands and expectations, especially those not feasible within the maternity ED, frequently trigger verbal aggression. Patients and their relatives may exhibit aggressive behavior by raising their voices, shouting, or demanding the immediate presence of physicians and emergency nursing care, even after receiving explanations regarding triage, prioritization, and waiting times. Emotional responses such as worries and anxiety often result from their perceived urgency and lead to expectations that their needs should be prioritized over those of others. As a result, they expressed unease and were doubtful about the outcomes of emergency care. These interactions appear to reflect not only frustration but also attempts to influence or expedite care through assertive or demanding communication. Nurses in the ED reported being either directly impacted or witnessing similar incidents among colleagues, including physicians. In most Gulf countries, female nurses predominantly provide maternity care nursing. Within this cultural context, physical aggression is restrained, as it is socioculturally denounced among women. Hence, verbal aggression is more commonly experienced than physical violence by female nurses in the ED.

“*We only encounter verbal…patients and their husbands shout, thinking that we will obey the… we will follow them*.” (P11)

“*I never experienced physical abuse. Their [patients and relatives] voices and actions I can understand. They want nurses to manage their patients right away, even if you explain the triage and that other patients are waiting. It is more of verbal abuse from the patients and relatives*” (P7)

“*At times they will just tap the table or chairs. They are just shouting. They raise their voice even if we try to explain to them. They want the doctor to come out and speak to them like that*.” (P9)

“*I was caring for a patient referred by EMS after completing the tasks for my previous patient. But the relative wanted to see the former patient, so I advised her to wait. Eventually, she started shouting at me, insisting on seeing her patient in the unit*.” (P8)

##### 4.2.1.2. Subtheme 1.2. Dissatisfaction With the Outcomes of Care

Dissatisfaction with nurses’ explanations or management, particularly regarding labor pain, frequently leads to escalation and verbal aggression. Patients and relatives often became distressed when expectations for immediate relief or intervention were not met. As a result, shouting and demanding behavior by the patients and relatives occurs. While communication was commonly identified as a contributing factor, the nurses’ accounts suggest that unmet expectations and anxiety about the patient’s condition also played a role in shaping response.

“*Communication is essential. Some, when not satisfied with our explanation, wait for the doctors to explain, and they will start shouting*.” (P1)

“*If the patient is in pain [labor pain], we can give medications, and other things we can do. However, if not relieved, they become alarmed and start to become anxious, demanding care. You need to educate them about managing pain and the specific case of their patient. Sometimes they demand doctors to do this*” (P6)

“*You have to communicate that you are a nurse. Treat the patients and relatives fairly. You have to communicate and be treated as a professional. Then some relatives will be relaxed and trust you as a nurse*” (P5).

“*You have to be polite so they will be polite. Sometimes they are not inquiring why the patient and relatives become violent. You can approach them politely and ask why they are shouting. Then, surely, they will explain and will be okay. From being violent, they become non-violent.*” (P10)

“*We need to communicate properly so they will understand. However, others don’t understand, so they expect differently. They become dissatisfied and start shouting at us. But good communication is the way to prevent WPV*” (P12)

Although participants emphasized respectful communication as a strategy to reduce aggression, these findings also indicate that dissatisfaction may stem from differences in expectations and understanding of care processes, rather than communication alone. Notably, despite cultural and religious norms emphasizing respect, participants reported persistent verbal aggression, highlighting a tension between expected demands for nursing care and social values and behaviors in high‐stress clinical situations.

#### 4.2.2. Theme 2: Sociocultural Differences Influence Nurse–Patient Interaction and Emergency Nursing Care

Language differences and cultural diversity appeared as key factors influencing interactions between nurses and patients and their relatives in the maternity ED. The sociocultural traits of patients and nurses influence communication, expectations, and responses in emergency nursing care.

##### 4.2.2.1. Subtheme 2.1. Language as a Barrier to Mutual Understanding

Language barriers were frequently identified as a challenge in nurse–patient communication. This occurs in a multicultural environment where staff and patients speak different languages and have diverse cultural backgrounds. Nurses reported that differences in language, beliefs, and communication styles often limited mutual understanding and contributed to tension during care encounters.

“*Understanding is the main thing. So, we have to understand each other because we come from different nationalities. The way we explain matters, so it is not easy to standardize everything*”. (P2)

“*We don’t totally know each other as we are different people with different languages, beliefs, and thoughts. Therefore, it would be better if we spoke a common language and acted professionally, as we come from various countries and have other nationalities”* (P9)

“*She [relative of the patient] shouted at me and wanted to speak to my supervisor. I called my colleague, but she is of the same nationality as me. So, she doesn’t want to talk to us both because of the language barrier*.” (P8)

“*Most new staff have problems because they don’t know the Arabic language. In the front line, there is Arabic-speaking staff in charge. The security can explain well to the patients because they speak Arabic. Security is helpful when there is a language barrier. However, security should also respond immediately upon noticing violence in the ED. We inform them to please come when we need help*.” (P14)

These challenges suggest that communication difficulties extend beyond language alone, reflecting broader differences in expectations and interpretation of care. Differences without mutual understanding may contribute to frustration and WPV.

##### 4.2.2.2. Subtheme 2.2. Diverse Representation Resolves Conflict

Linguistic and cultural diversity within the workforce, supported by a positive attitude toward inclusivity, can mitigate misunderstandings and violence, which is very common in high‐stress units like ED. Nurses who are inclusive and have adapted to patients’ and their relatives’ linguistic or cultural backgrounds can deliver culturally congruent care, which can de‐escalate emotionally charged behaviors in the ED. For instance, patients and relatives rely on Arabic‐speaking staff when they speak Arabic. Thus, representation within a diverse workforce also enhances nurse–patient interactions and helps prevent miscommunication. Diversity, when leveraged effectively, enhances communication and trust between the nurses and the patients and their relatives. Thus, both diversity and inclusivity can be identified as active WPV protective factors that bridge the linguistic gaps and ensure culturally congruent care.

“*There are situations when someone is aggressive, like shouting, my fellow nurse often calls me for help because I know the Arabic language*.” (P4)

“*Once they raise their voice, you cannot pacify them. So, I explained the situation to our charge nurses, but others need Arabic-speaking staff, so we will call the security who are good at it*.” (P7)

These findings suggest that language proficiency is essential for clear communication and high nurse–patient interaction. The presence of linguistically aligned staff reduces frustration and facilitates timely responses to patient needs. In Qatar’s diverse community, an inclusive approach within ED enhances cultural understanding and ensures culturally congruent care. Thus, communication and inclusivity reduce misunderstandings and build trust. These also act as active protective factors that help prevent conflict and de‐escalate verbal aggression.

#### 4.2.3. Theme 3: Misaligned Expectations and Policy Awareness

Limited awareness of hospital procedures and policies emerged as a key factor contributing to WPV. Lack of awareness or misunderstandings around triage, admission, and care processes often led to unmet expectations and conflict.

##### 4.2.3.1. Subtheme 3.1. Nonadherence Due to Limited Awareness of the Hospital Guidelines

Nurses reported that limited awareness and understanding of ED procedures and the cases seen for tertiary care services contributed to dissatisfaction and verbal aggression by the patients and relatives. They lack awareness about triage, admission, and other operational processes in the ED. They become impatient with challenges, such as delays in response, prioritization, and visitation. This behavioral response stems from a misalignment between their own understanding and the staff’s perception and awareness of hospital policies.

*“They want to be in here (maternity facility) compared to primary health centers because they are more satisfied and feel safe. They insist on being here since the services are better. The patients should be educated and oriented about the process in ED.”* (P2)

*“It will take 30 minutes, but up to 1 hour and 30 minutes for the ultrasound procedure and results. They become irritated, shouting, and will not go back to the ultrasound”* (P6)

*“We are deciding based on priorities like those in either 1, 2, or 3. It only gets delayed when it is really too busy, like bleeding patients, IVF patients, and those about to deliver their baby are all coming together in the ED at the same time. Patients and relatives cannot wait a long time to see the doctor, which triggers aggression.”* (P8)

*“We can inform them about the policy, especially the consequences of WPV. We have to know what we can and cannot do, so we have confidence in how to stop WPV.”* (P12)

*“If they are informed that we receive emergency cases, then we can manage easily. If they are publicly aware, this may help avoid unnecessary visits to the ED and reduce the waiting time for patients who are eligible for emergency care and admission.”* (P6)

*“The patients and the community should be informed. They should be aware of WPV and its sanctions. There should be mutual understanding between staff and patients, as well as among staff members. It’s also good to initiate self-learning and search for updates and polices in WPV.”* (P13)

*“Due to the setting, we don’t allow male visitors inside the ED. They can be in the waiting area. Additionally, most guests arrive in groups, often due to traditional or cultural affinities that provide family support. However, this is also not permitted according to hospital policy in the ED. We are explaining, though some understand, but in instances, this leads to shouting as well.”* (P15)

These accounts suggest that gaps in awareness not only affect understanding of care processes but also shape how patients interpret delays and restrictions, often leading to conflict when expectations are misaligned with hospital policies.

##### 4.2.3.2. Subtheme 3.2. Patients’ and Families’ Expectations Override Hospital Policy

Despite the availability of guidelines, signage, and staff explanations, nurses reported that patients and relatives do not always adhere to hospital procedures, particularly when their expectations were unmet. When regarded as emergencies, their request for emergency nursing care must be responded to immediately due to their perceived urgency and fear. This often conflicts with hospital policies and procedures, resulting in disagreements and escalation.

“*If we can show them the guidelines, then we have the confidence to correct them. Too much patience can cause stress, and stress leads to violence. If we have enough staffing, patients will not wait any longer.*” (P1)

“*If, according to their demand, we do not provide the care, patients and relatives become violent. If they want to use some isolation area and we are restricting them, they will eventually argue with us*.” (P1)

“*They are saying that if the delay harms the baby, who will be held responsible. They are informed while sitting in the waiting area about triage and prioritization, but they will argue when they are waiting for a long time and sometimes won’t listen to us anymore*.” (P3)

“*When we explain that we are doing tasks and so advise them to wait, the shouting starts in here. There are two frontline nurses in the ED. One will bring the patient to the isolation room when needed. The other one will take in patients in labor and other related cases. So, one will cover the other, which they don’t know or understand. They all wanted to be a priority since they went to the ED*.” (P13)

“*We are deciding based on priorities like those in either 1, 2, or 3. It only gets delayed when it is really too busy like bleeding patients, IVF patients, and those about delivery her baby are all coming together in ED at the same time. Patients and relatives cannot wait a long time to see the doctor*.” (P8)

“*We explain to the patient about the duration of the procedures. Others wanted this fast, but there are guidelines that we need to follow. We mention that the time will be a little bit longer, and they get angry and shout.*” (P14)

These findings indicate that awareness alone may not ensure compliance, as adherence to guidelines appears to be influenced by patient expectations, perceived urgency, and tolerance for waiting.

#### 4.2.4. Theme 4: Nurses’ Adaptation and the Competing Care Demands

The maternity ED environment was described as a high‐pressure setting characterized by high patient volume, multiple urgent cases, workflow disruptions, and delays in prioritization and treatment. In addition, both competing care demands and complex patient assignments trigger WPV.

##### 4.2.4.1. Subtheme 4.1. System Pressure From Competing Care Demands

Competing care demands are frequently identified as a substantial factor in WPV. Nurses reported situations in which multiple patients required immediate attention simultaneously. When patients were asked to wait, this often led to frustration and escalation among patients and their relatives.

In such situations, patients and their relatives perceived their need for emergency care as urgent. They added that delays in emergency nursing care are often viewed as a negative hospital experience.

*“The pattern we are seeing in the emergency is that they all consider a priority, so when the frontline nurse advises them to wait as she will attend to the first patient, it begins there, one starts to raise their voice, and others will come together.”* (P2)

*“With relatives like mother and sister, violence will occur more. Sometimes we become busy, and we don’t get times to see other patients at that time. Especially when they request doctors, this makes them violent”* (P6)

“We have staff, but the ratio of about 8‐10 patients per shift is a heavy workload. So, all things are fast, even breaks. The demand for patient care is high for the patients and their relatives.” (P10).

*“When the workflow is followed correctly, like treating and approaching patients according to plans, we don’t have much violence. However, if the flow is interrupted, as is often the case when many patients arrive, and there is crowding, then we cannot treat them one at a time. So, dissatisfaction happens, and yeah, violence occurs.”* (P15)

These accounts suggest that WPV is closely linked to workflow disruptions, perceived delays, and mismatches between patient expectations and service capacity within the ED.

##### 4.2.4.2. Subtheme 4.2. Resilience of the Emergency Nurses

Exposure to WPV can be frustrating and cause emotional stress. However, this study found that nurses respond with adaptive coping strategies to sustain nursing care in the ED. These included multitasking, prioritizing high‐risk patients, seeking support from colleagues, and maintaining focus on clinical responsibilities despite experiencing verbal abuse. Some nurses also describe emotional coping mechanisms, such as verbalizing their experiences with others for support or distancing themselves from stressful interactions to reduce the emotional impact of WPV.

“*We need to multitask in an emergency, as we need to complete the benchmarks. I see 2-3 patients at a time, especially during rush hours. Staff should help each other, especially for those having high-risk patients, so that we can attend to all of them.*” (P2)

“*Feels really bad when they shout, but we still have to do our job. We cannot leave our patients. Shouting should not affect patient care*.” (P6)

“*I convey my feelings to my colleagues. Even at home, I also share with my husband and somehow pray as well. This happens when they say I am responsible for what happens to their baby*.” (P3)

“*You have to take steps or distance a bit before it will lead to maybe physical abuse. I tell my charge nurse after reporting that tomorrow is just another day. Some patients appreciate those who offer simple gestures and express their gratitude. It reminds you why you took up nursing, and it’s nice to be a nurse.*” (P5)

The nurses exert efforts to sustain care despite WPV and manage emotional stress while continuing to function with the demands. These strategies indicate resilience, making them adaptive and perceptive regarding WPV as a common anticipated experience when assigned in the ED.

#### 4.2.5. Theme 5: Managing WPV With Manpower Resources and Collaboration

Nurses described the importance of appropriate manpower, collaborative approaches, and peer‐support systems in responding to WPV in the ED.

##### 4.2.5.1. Subtheme 5.1. Workforce Crisis Increases Risk to WPV

Workforce‐related challenges, such as staff shortages and heavy workload, were frequently identified as contributing factors to WPV. Nurses described how increased workload and limited staffing often lead to longer wait times, which intensify patient disappointment and increase the likelihood of aggressive behavior.

*“… support from the security is needed as there are other things that are beyond our control.”* (P11)

*“Charge nurses and head nurses help us if we cannot control the shouting. We involve them, and they help us.”* (P1)

*“This shortage of staff … less staff and fewer doctors, so patients are waiting, and we cannot quickly solve their problems so that the violence will be more”*. (P1)

*“When it happens, I’m leaving this and not taking that to my home. I will feel it, but after I’m trying to cope.”* (P6)

*“I cannot say a person is the reason for WPV in ED, but the staff shortage. Sometimes the doctors are reassigned more to the ED if patient numbers are increasing. Shortage is the cause, because if the staff is overloaded, then the patient needs to wait more, which leads to violence”* (P12)

These findings indicate that workload levels and the availability of an experienced workforce not only affect service delivery but also shape the frequency and management of WPV within the ED.

##### 4.2.5.2. Subtheme 5.2. Team‐Based Response for Safety and Managing Aggression

Peer support from colleagues, supervisors, and security staff was also highlighted as necessary in managing WPV. Thus, team‐based approaches provide emotional reassurance and have been described as important for managing and de‐escalating WPV in the ED. Nurses highlighted the importance of teamwork, coordination, and immediate support from colleagues and security staff in managing aggressive situations and ensuring safety.

“*We need good support so this WPV will be controlled*.” (P2)

“*If we work as a team, we can do everything easily. In all emergencies, we need a good team. If two or three people work together, it will be easier. If other staff, including managers, will receive the patient or secure beds in preparation for patient transfer while I’m busy, then that will help me a lot*.” (P6)

“*Teamwork is needed, and saying apologies can solve these challenges in ED*.” (P9)

“*Availability of security is essential. They [security] ensure safety and security. We feel at ease, more protected, and that someone is there for me, especially during busy times*.” (P5)

These findings suggest that collaborative efforts provide both practical and emotional support for nurses, helping them manage challenging interactions and maintain a sense of safety in the workplace.

## 5. Essence

**FIGURE 1:**
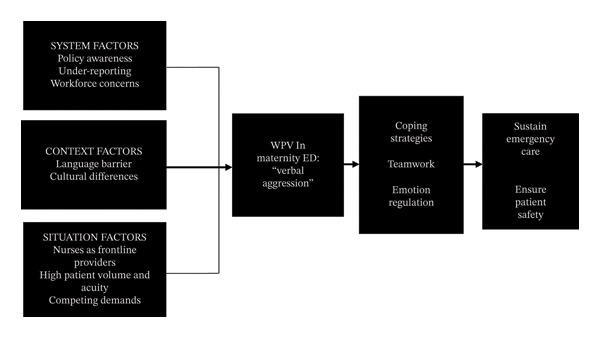
The WPV conceptual model depicts multilevel drivers resulting in verbal aggression, which nurses respond to with coping strategies, teamwork, and emotion regulation to sustain nursing care and safety.

As shown in Figure 1, WPV in ED has multilevel drivers that can be clustered as system‐based, situation‐grounded, and contextual. Another key insight is that verbal aggression is the most commonly reported WPV manifestation by the patients and their relatives. ED nurses recognize verbal aggression as a frequent occurrence in the ED and perhaps linked with sociocultural norms as seen in maternity settings. In Arab countries, such as Qatar, the prevalence of verbal aggression reflects cultural boundaries that limit physical forms of violence, particularly in interactions with female healthcare workers, such as maternity ED nurses.

These findings provide a clearer picture of WPV in maternity EDs. WPV often occurs due to misunderstandings, unmet expectations, communication problems, and a lack of awareness of hospital policies. In Qatar, where the workforce and community are diverse, strong language skills and inclusive practices help build trust, improve staff communication, and reduce the likelihood of emotional reactions from patients and their families. Challenging working conditions, such as heavy workloads, staff shortages, and workflow interruptions, can lead to delays and longer waiting times, which may result in WPV in ED. Staff who are culturally sensitive and communicate well with patients and families can help close these gaps and calm situations before they turn into verbal aggression. Nurses say that facing WPV is now common, but this experience has helped them learn more about WPV, develop coping strategies, value teamwork, and manage their emotions while providing care. The flexibility and commitment of nurses in emergency services help maintain patient care and ensure safety, demonstrating how WPV in the ED among nurses builds their professional resilience.

## 6. Discussion

This study explored the lived experiences of maternity nurses regarding WPV in the ED in a tertiary maternity hospital in Qatar. The findings demonstrate that verbal aggression, sociocultural differences, limited policy awareness, the demanding emergency culture, and safety concerns are interconnected factors shaping nurses’ experiences. Rather than occurring as isolated incidents, WPV appears to arise from the interaction between emotional strain, cultural complexity, and organizational pressures within a multicultural maternity emergency environment.

Verbal aggression emerged as a central and recurring aspect of nurses’ experiences, identified as the most frequent and distressing form of WPV. Encounters involving shouting, harsh language, and unreasonable demands, often triggered by dissatisfaction with waiting times or perceived delays, were common [25]. Consistent with global evidence identifying verbal abuse as the most prevalent form of WPV in emergency settings among female nurses [26, 27], these findings suggest that such behavior extends beyond a simple reaction, reflecting patients’ attempts to regain control in situations characterized by uncertainty, pain, and perceived urgency.

Nurses reported feelings of humiliation and reduced confidence, reinforcing evidence that verbal abuse undermines self‐esteem and performance [20]. Over time, repeated exposure contributed to emotional fatigue and normalization of such behavior, which may help explain persistent under‐reporting observed in this and other studies [21, 28, 29]. This normalization indicates that WPV extends beyond individual encounters, reflecting broader systemic pressures within emergency care environments. It also appears to influence nurses’ professional roles and interactions. Repeated exposure to verbal aggression may lead to emotional distancing and a shift toward more task‐focused care, as nurses adopt protective strategies to sustain functioning in a high‐pressure environment. Over time, this may reshape how nurses engage with patients, moving from relational care toward more functional approaches.

The maternity ED in Qatar is home to a wide range of cultures and languages, which makes nurse–patient interactions more emotionally charged. Nurses say that language barriers, different expectations, and varying views on care often cause miscommunication and tension, which sometimes lead to WPV. Most nurses are expatriates from diverse backgrounds, while patients also come from many cultures, including Arabic‐ and non–Arabic‐speaking patients. This difference often causes communication problems and can make some patients hesitant to accept care from nurses who do not speak their language. The study shows that nurses’ language skills and having staff who reflect the patients’ ethnic backgrounds help improve nurse–patient interactions. Better language abilities and a more culturally representative staff can set clearer expectations. This mechanism reduces anxiety for everyone, prevents conflict, builds trust, and supports care that fits patients’ cultures, thereby lowering the risk of WPV [30].

Within this context, nurses may experience a sense of professional disempowerment, as cultural and linguistic differences shape perceptions of their roles and authority. This may contribute to situations in which care is questioned or refused, increasing tension and the likelihood of verbal aggression and ultimately undermining a safe and respectful care environment. While previous studies identify sociocultural diversity as a contributing factor to WPV [31, 32], the present findings suggest that these differences shape not only communication, but also how care is perceived and evaluated by patients and families. In this context, misunderstandings may reflect deeper gaps in expectations regarding urgency, entitlement to care, and trust in healthcare providers. This helps explain why, despite explanations, some patients continued to escalate their responses. Nurses’ reliance on multilingual colleagues highlights the importance of cultural mediation, suggesting that effective communication in such settings requires not only language proficiency but also cultural interpretation and valuing differences [30, 33, 34].

Limited awareness of hospital policies related to workplace behavior and reporting procedures contributed to WPV. Nurses described how misunderstandings related to triage, waiting times, and access restrictions often lead to frustration and conflict. While previous studies link unclear policies to increased violence [35–37], the current findings suggest that the issue extends beyond awareness alone, pointing instead to deeper expectations surrounding urgency and access to care. These findings align with evidence that system‐related factors, such as long waiting times and understaffing, can contribute to patient frustration and a sense of being unheard in emergency settings. In such high‐stress, unfamiliar environments, patients may prioritize their own needs and perceive delays or restrictions as neglect or threats to the quality of care, thereby increasing the likelihood of aggressive behavior toward healthcare staff [38]. This insight is further supported by cultural understandings of waiting in healthcare settings, as described by Hassankhani et al. [38], in which patients may adopt a “me first” approach, prioritizing their own needs over others. These findings are particularly relevant in environments characterized by uncertainty and prolonged waiting times. This perspective aligns with current findings that patients and their relatives often expect immediate attention and become frustrated when delays occur. Such behavior may reflect not only dissatisfaction but also culturally influenced expectations of care, where waiting is perceived as a threat to safety or quality, thereby increasing the likelihood of verbal aggression. Rather, these incidents reflect a mismatch between institutional processes and patient expectations, in which delays or restrictions are perceived as neglect or inadequate care. This highlights the importance of understanding WPV not only as an individual behavior but as a culturally and contextually shaped response to perceived delays and unmet expectations.

The fast‐paced and demanding nature of the ED environment further amplified these challenges. Overcrowding, delays, and emotional strain increased frustration among patients and their families, often leading to escalation [39]. In maternity settings, where care is inherently family‐centered, the presence of multiple relatives and heightened emotional involvement may further intensify these dynamics [40]. These findings suggest that WPV is closely linked to structural pressures within the ED, where competing demands and staff shortage create conditions in which expectations cannot always be met. Over time, repeated exposure to such situations contributed to the normalization of aggression, with nurses perceiving it as an inevitable part of their role, a pattern also documented in a previous study [34]. This normalization may have important implications, including reduced reporting, acceptance of unsafe conditions, and emotional disengagement from patient interactions. Safety concerns were consistently reported, with nurses describing feelings of vulnerability, particularly during high‐demand periods or when support was limited. These experiences align with global evidence linking WPV to burnout, emotional exhaustion, and reduced job satisfaction [19, 41–43]. In response, teamwork emerged as a key protective factor, providing both practical assistance and emotional support. This suggests that collaborative environments not only improve operational efficiency but also enhance nurses’ sense of security and resilience. Similar findings have been reported by Babaei et al. [44], highlighting the role of shared responsibility and collective coping in managing WPV. These findings reinforce the importance of strengthening team‐based approaches and support systems as part of comprehensive strategies to address WPV in ED.

Taken together, these findings demonstrate that WPV in maternity ED settings is shaped by a complex interaction of emotional, cultural, and organizational factors. Rather than isolated incidents, WPV reflects broader systemic and contextual challenges that influence both patient behavior and nurses’ experiences. Addressing these issues requires coordinated efforts that extend beyond policy implementation to include cultural understanding, effective communication, workforce support, and collaborative practices. Such efforts may not only reduce WPV but also enhance the overall quality and safety of care [34, 43, 45].

At the organizational level, the action plan includes reinforcing existing policies and ensuring their consistent implementation. In the Qatari context, national frameworks such as SA 1058, SA 1004, and HR 3047, along with structured training programs, provide a strong foundation for improving safety and staff security. Expanding public awareness initiatives, enhancing staff training in conflict management, and promoting clear communication of patient rights and responsibilities may further support efforts to reduce WPV in maternity ED settings [46, 47].

### 6.1. Limitations

The study’s findings have limited generalizability as these are based on 15 female nurses from a single maternity center within the corporation’s four facilities. The bases of the findings and conclusions are the nurses’ narratives, which are subjective interpretation of the phenomenon, thereby restricting how broadly they may be applied. However, the exploration was more focused on the lived individual experience of maternity nurses, providing a more in‐depth understanding of WPV as a phenomenon in maternity care clinical settings.

## 7. Conclusion

Examining the lived experiences of maternity ED nurses provided deeper insights and unraveled the mechanisms of WPV within tertiary maternity setting. Overall, the WPV in the maternity ED is a multilevel phenomenon involving system controls, situational strains, and sociocultural factors. Also, workforce diversity promotes both ethnic representation and language proficiency, which are essential for high‐quality nurse–patient interactions. Language proficiency and inclusive practices enable nurses to provide culturally congruent care. This approach supports clearer communication, aligns expectations, and fosters trust, particularly within diverse communities. These factors and mechanisms are critical in preventing and de‐escalating WPV.

Based on the findings, healthcare organizations and nursing managers can address this issue by promoting participation in training focused on language proficiency, teamwork, and aggression management. Previous literature indicates that interprofessional collaboration fosters a supportive and positive work environment. Thus, collaborative action planning and the implementation of preventive measures are recommended to prevent and de‐escalate WPV in the ED. Administrators are recommended to encourage staff to utilize reporting systems and provide prompt responses and feedback. For example, the use of standardized emergency alerts with assigned codes can facilitate rapid unit‐ or facility‐based responses. Raising public awareness of ED culture, operational procedures, and the legal implications of WPV through media and social engagement platforms is recommended. Overall, prevention and de‐escalation of WPV can be achieved by implementing context‐specific policies, providing support and education, and enhancing collaboration to protect employees and stakeholders in the maternity ED.

## Author Contributions

A.S.A., J.P.B.S., G.C., R.D.L., L.P., S.M., S.A.V., and D.H.D.J.: the conception and design of the study.

A.S.A., J.P.B.S., G.C., and R.D.L.: acquisition of data.

A.S.A., J.P.B.S., A.M.A.A., and D.H.D.J.: data analysis and interpretation of data.

A.S.A., J.P.B.S., A.M.A.A., G.C., R.D.L., L.P., S.M., S.A.V., D.H.D.J., R.R.M., and R.A.S.I.: drafting the article or revising it critically for important intellectual content.

A.S.A., J.P.B.S., A.M.A.A., G.C., R.D.L., L.P., S.M., S.A.V., D.H.D.J., R.R.M., and R.A.S.I.: final approval of the version to be submitted.

## Funding

Open access funding was provided by the Qatar National Library.

## Ethics Statement

The Institutional Review Board approval was obtained from the Medical Research Center of Hamad Medical Corporation in Doha, Qatar (MRC 01‐21‐817). The study was conducted in full conformance with the principles of the “Declaration of Helsinki” and Good Clinical Practice (GCP) and within the laws and regulations of the Ministry of Public Health in Qatar.

## Conflicts of Interest

The authors declare no conflicts of interest.

## Data Availability

Data supporting the findings of this study are available from the corresponding author upon reasonable request.

## Appendix A Questions in the Semistructured Interview Guide

1. How would you describe your experience of workplace violence in ED?
2. Can you describe how you coped with the WPV experience?
3. Have you witnessed workplace violence in ED? If yes, can you describe what happened?
4. In your experience, who is/are causing WPV in ED?
5. What do you think are the causes of WPV, and why are they doing it?
6. What do you think are the consequences of WPV to the staff, patient care, and working environment?
7. What strategy do you use to prevent WPV in ED?
8. If there are people or a person who reminds you of WPV, who are they or he/she? Why does it remind you of WPV?
9. Can you tell more about an area or workplace in ED that reminds you of WPV in ED? Why does this area remind you of WPV?
10. Can you tell more other people, or things that remind you of WPV in ED?
11. If you were invited to speak about WPV in ED, as a maternity nurse, what would be your key message, and why?
